# Supplementary material for: Hydrogen spillover-driven synthesis of high-entropy alloy nanoparticles as a robust catalyst for CO2 hydrogenation
Source: Nat Commun. 2021 Jun 23;12:3884. doi: 10.1038/s41467-021-24228-z (PMC8222268; doi:10.1038/s41467-021-24228-z)
Supplement: Supplementary file 1 — Supplementary information [file 41467_2021_24228_MOESM1_ESM.pdf]

Supplementary Information

Hydrogen Spillover-Driven Synthesis of High-Entropy Alloy Nanoparticles as a Robust Catalyst for CO_2 Hydrogenation

Kohsuke Mori[1,2,3]*, Naoki Hashimoto[1], Naoto Kamiuchi[4], Hideto Yoshida[4], Hisayoshi Kobayashi[5], and Hiromi Yamashita[1,2,3]*

[1] Division of Materials and Manufacturing Science, Graduate School of Engineering, Osaka University, 2-1 Yamada-oka, Suita, Osaka 565-0871, Japan.

Tel & FAX: +81-6-6879-7457, +81-6-6879-7457,

E-mail: mori@mat.eng.osaka-u.ac.jp, yamashita@mat.eng.osaka-u.ac.jp

[2] Elements Strategy Initiative for Catalysts Batteries ESICB, Kyoto University, Katsura, Kyoto 615-8520, Japan.

[3] Innovative Catalysis Science Division, Institute for Open and Transdisciplinary Research Initiatives (ICS-OTRI), Osaka University, Suita, Osaka 565-0871, Japan.

[4] The institute of Scientific and Industrial Research, Osaka University, 8-1Mihogaoka, Ibaraki, Osaka 567-0047, Japan.

[5] Kyoto Institute of Technology, Matsugasaki, Sakyo-ku, Kyoto, 606-8585, Japan.

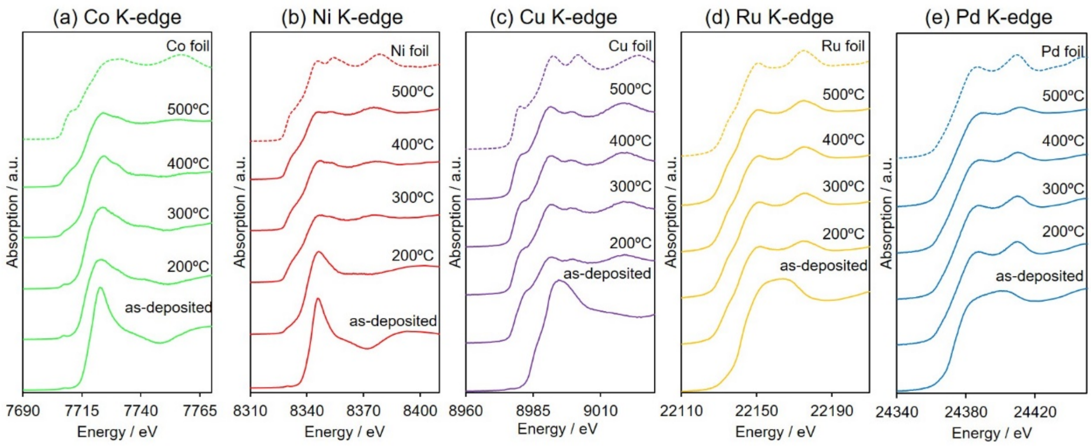

Supplementary Figure 1. In situ XANES spectra at the (a) Co, (b) Ni, (c) Cu, (d) Ru, and (e) Pd K-edge of CoNiCuRuPd/TiO_2 acquired during reduction under H_2 from room temperature to 500 ºC.

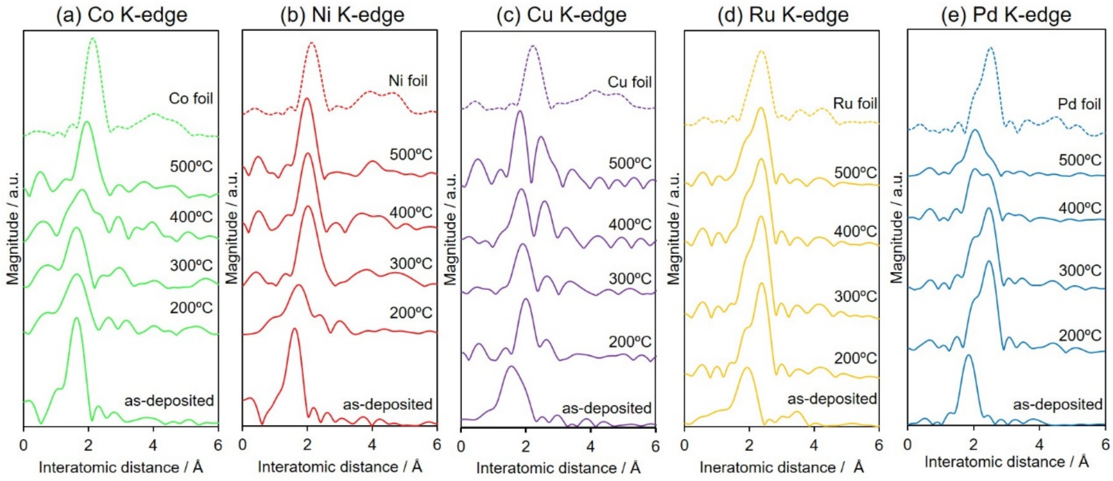

Supplementary Figure 2. In situ FT-EXAFS spectra at the (a) Co, (b) Ni, (c) Cu, (d) Ru, and (e) Pd K-edge of $CoNiCuRuPd/TiO_2$ acquired during reduction under H_2 from room temperature to 500 ºC.

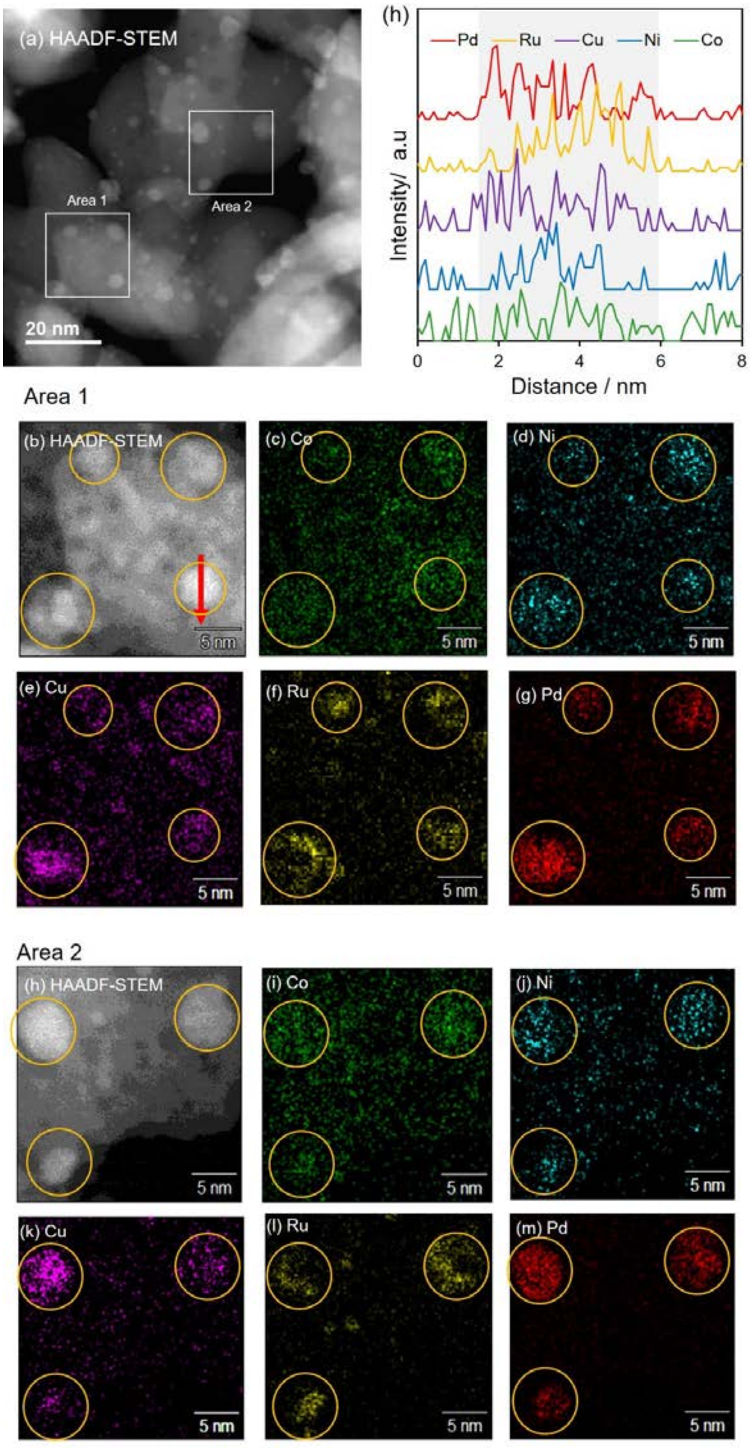

Supplementary Figure 3. Characterization of the $CoNiCuRuPd/TiO_2$. (a) HAADF-STEM image of $CoNiCuRuPd/TiO_2$, (b) HAADF-STEM image of Area 1, (c)-(g) EDX mapping of the corresponding elements, (h) EDX line analysis along the arrow in (b). (h) HAADF-STEM image of Area 2, (i)-(m) EDX mapping of the corresponding elements.

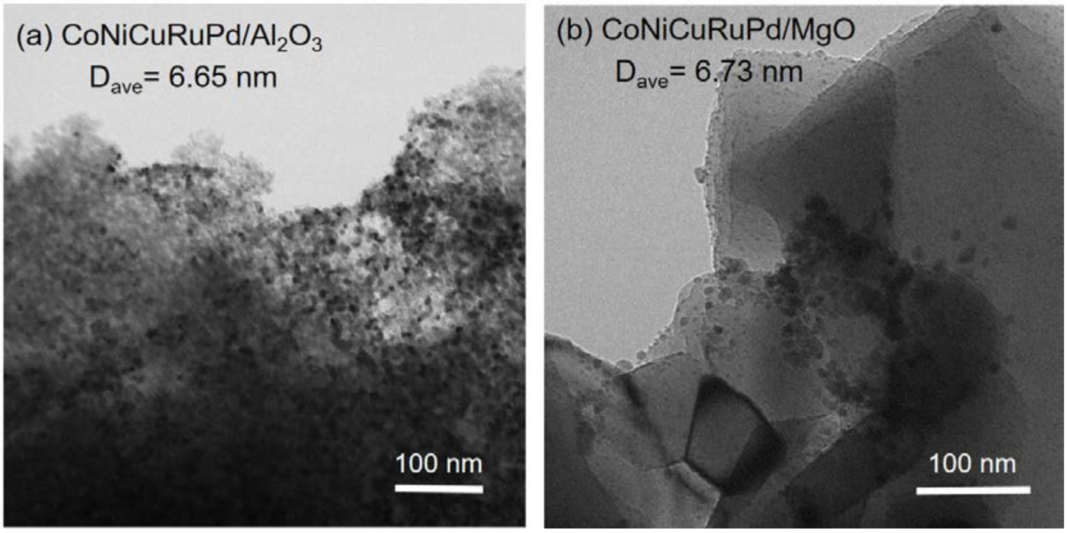

Supplementary Figure 4. TEM image of (a) CoNiCuRuPd/Al_2O_3 and (b) CoNiCuRuPd/MgO.

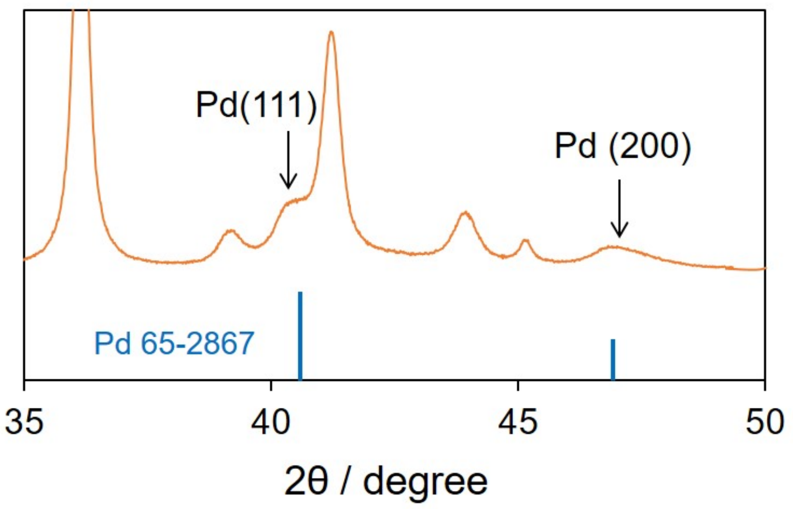

Supplementary Figure 5. In situ XRD pattern of CoNiCuRuPd/TiO_2 after reduction under H_2 at 200 ºC.

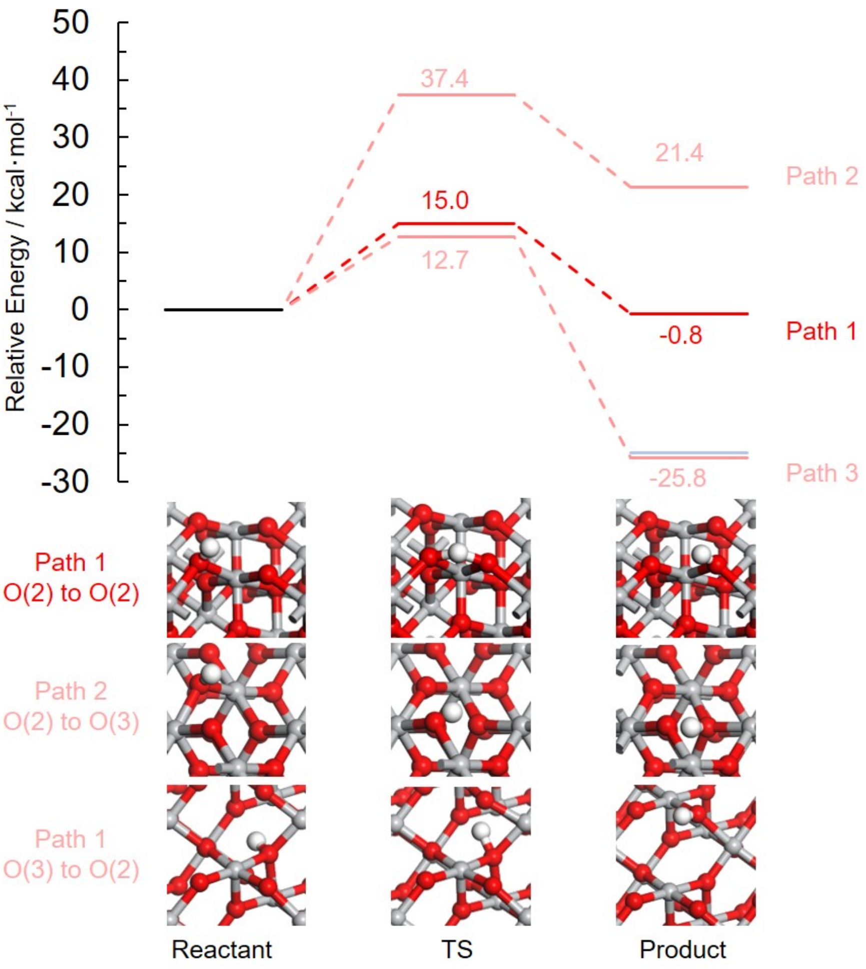

Supplementary Figure 6. Energy profiles and calculated model for the H atom migration (Step 3) on the TiO_2(101).

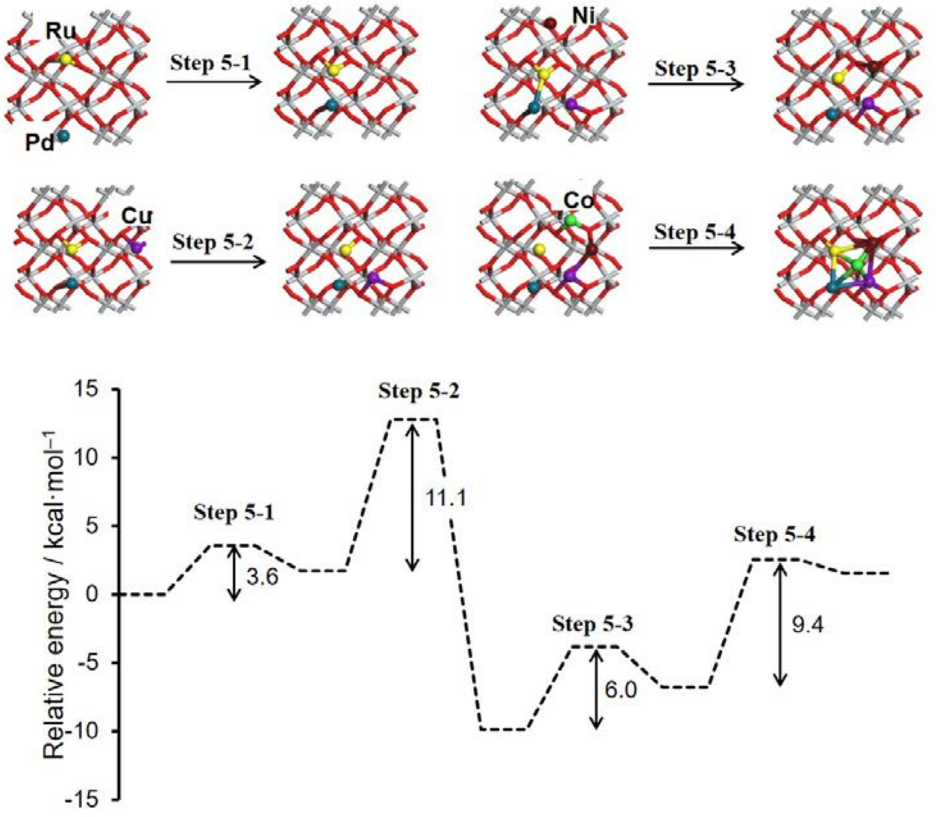

Supplementary Figure 7. The potential energy profile of **Step 5** (formation energy of 5-nucler cluster model containing 5 different elements from the reduced each atom) on the TiO2 (101) obtained by DFT calculations. The values are calculated energy barriers.

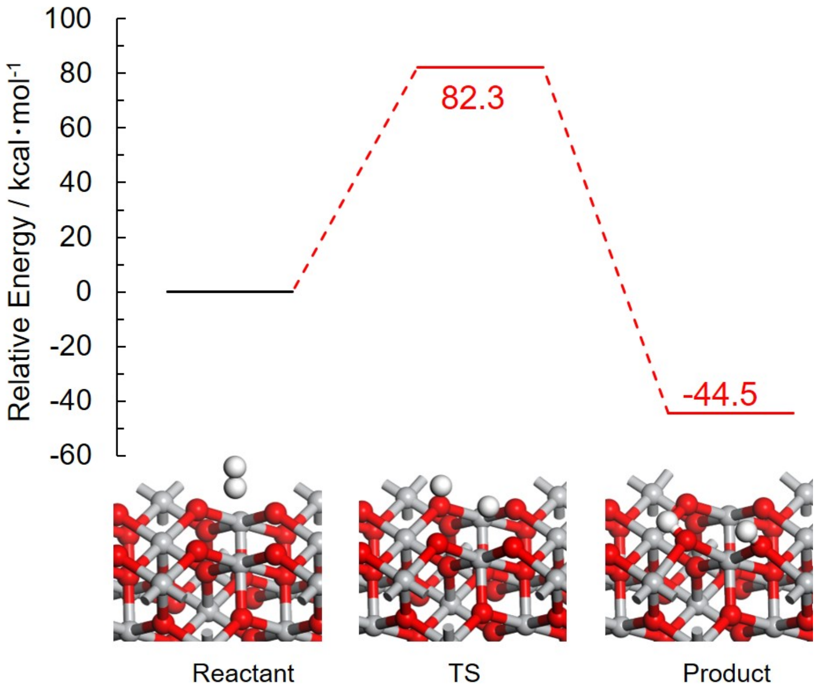

Supplementary Figure 8. Energy profiles and calculated model for the H_2 cleavage on TiO_2 (101) without Pd_5 cluster

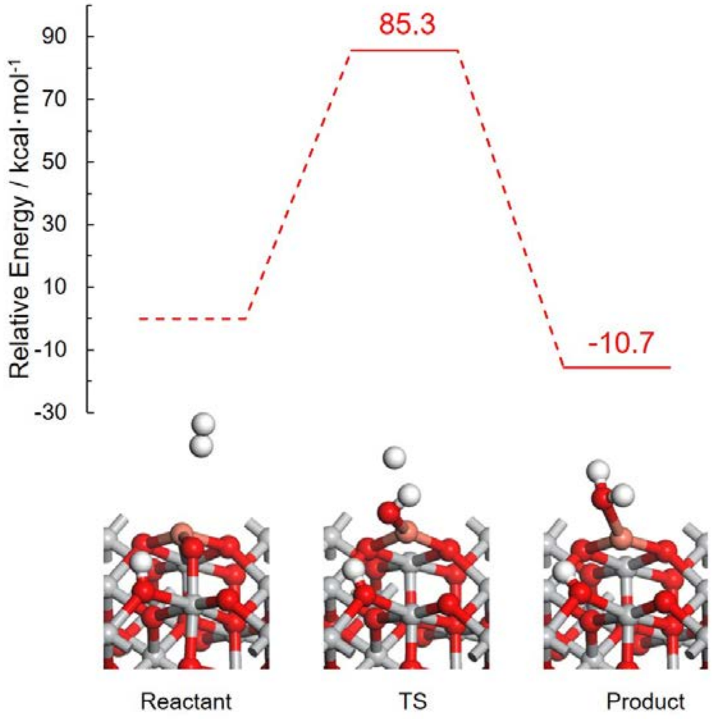

Supplementary Figure 9. Energy profiles and calculated model for the reduction of Co^{2+} species by vapor H_2 on the TiO_2.

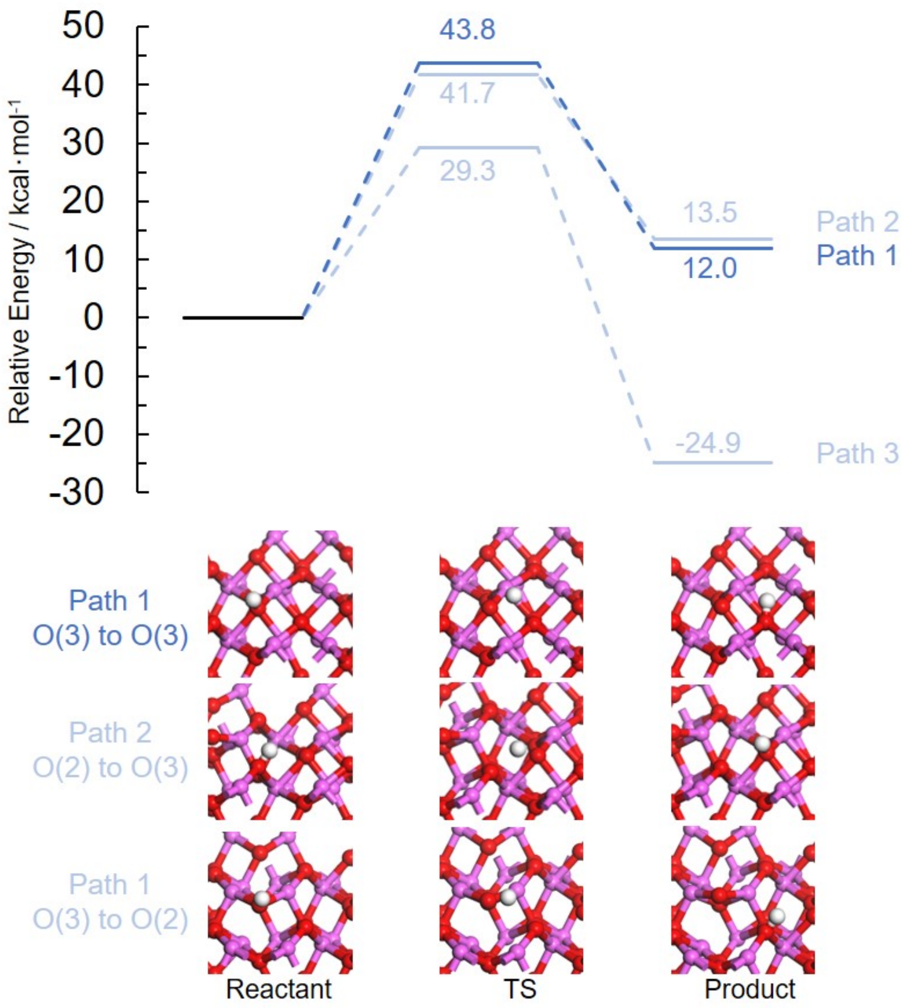

Supplementary Figure 10. Energy profiles and calculated model for the H atom migration (Step 3) on Al_2O_3(100).

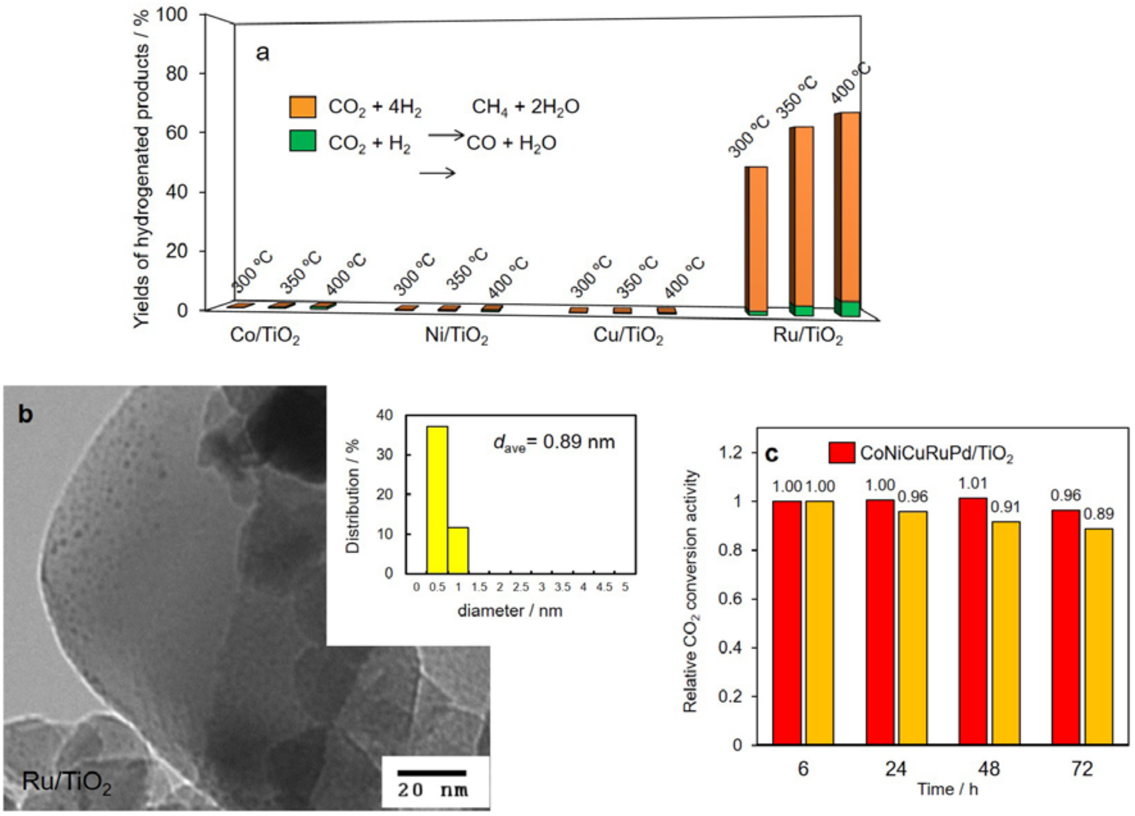

Supplementary Figure 11. (a) Yields of hydrogenated products over monometallic Co, Ni, Cu, and Ru-supported TiO_2, (b) TEM image and size distribution diagrams of Ru/TiO_2, and (c) relative activities over time, showing the durability of CoNiCuRuPd/TiO_2 and Ru/TiO_2 during CO_2 hydrogenation.

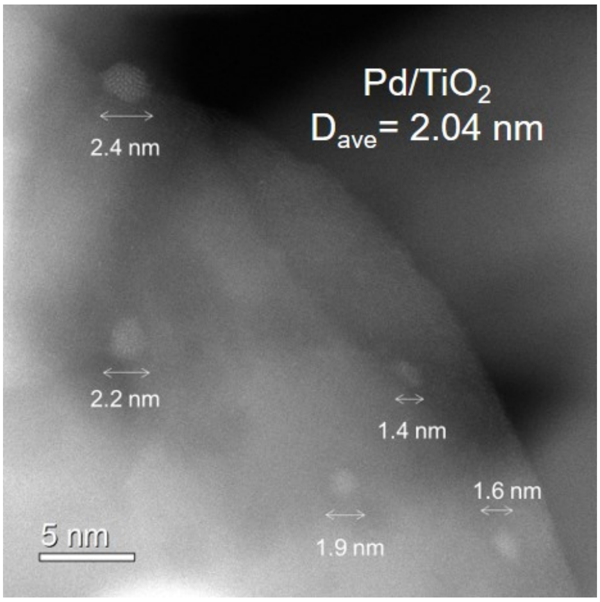

Supplementary Figure 12. STEM image of monometallic Pd/TiO_2

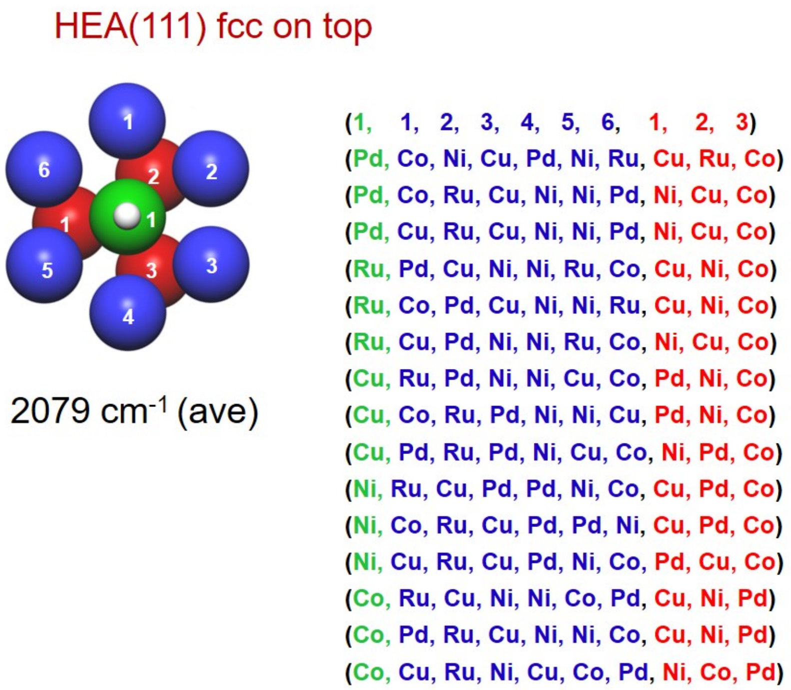

Supplementary Figure 13. *fcc* CoNiCuRuPd(111) facets of periodically repeated slab models for the calculation of on top adsorbed CO frequency. The employed 15 configuration patterns are depicted.

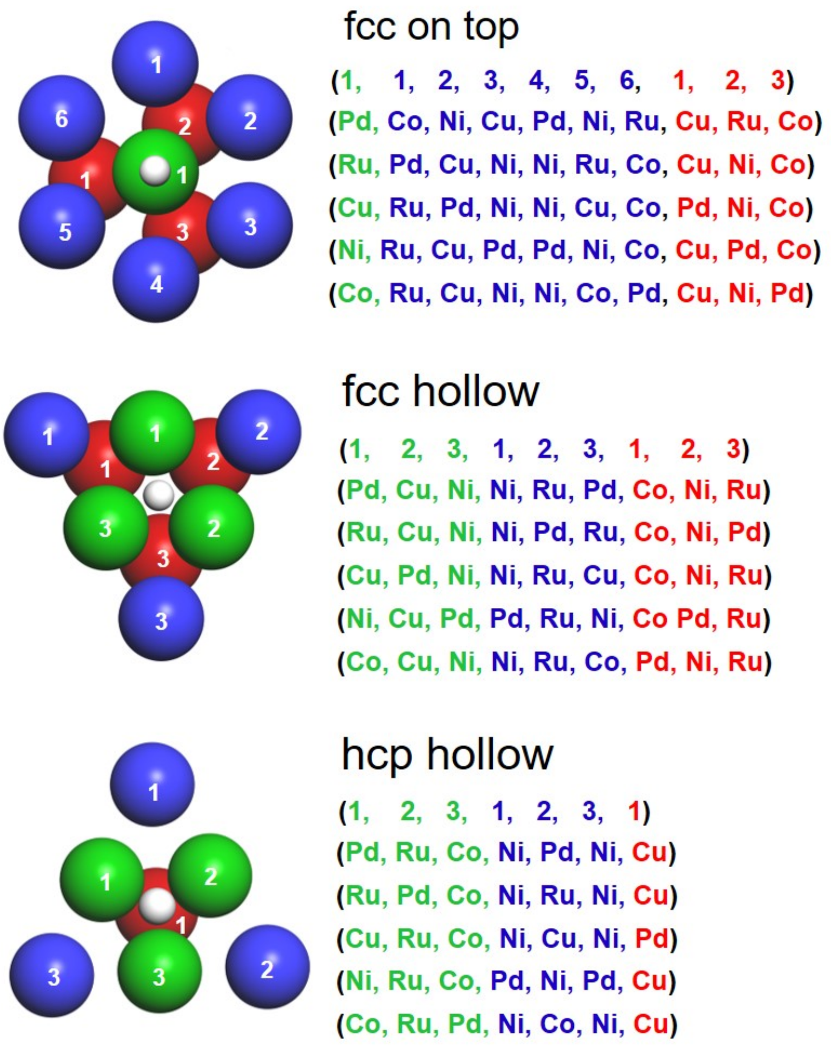

Supplementary Figure 14. CoNiCuRuPd(111) facets of periodically repeated slab models for the calculation of the adsorption energies (E_{ad}) of CO and H on fcc on top, fcc hollow, and hcp hollow. The employed 15 configuration patterns are depicted.

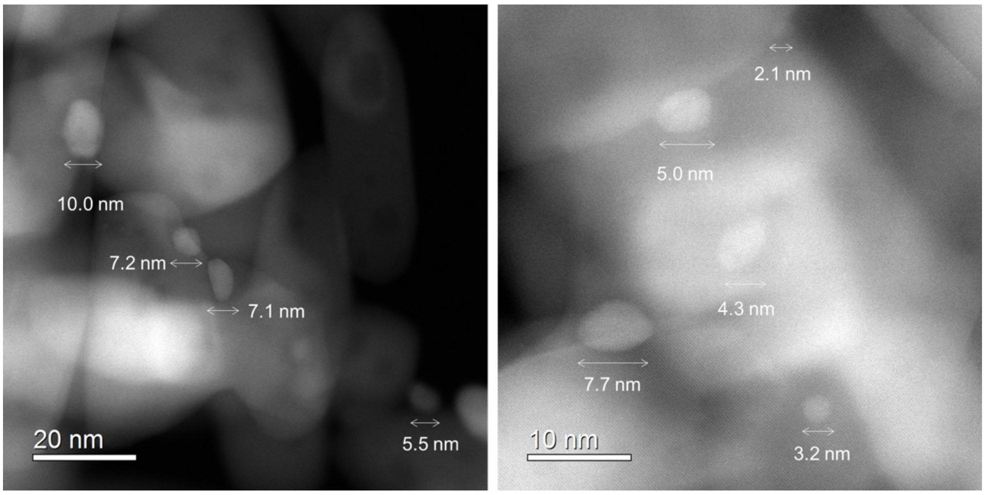

Supplementary Figure 15. HAADF-STEM images of used Pd/TiO_2

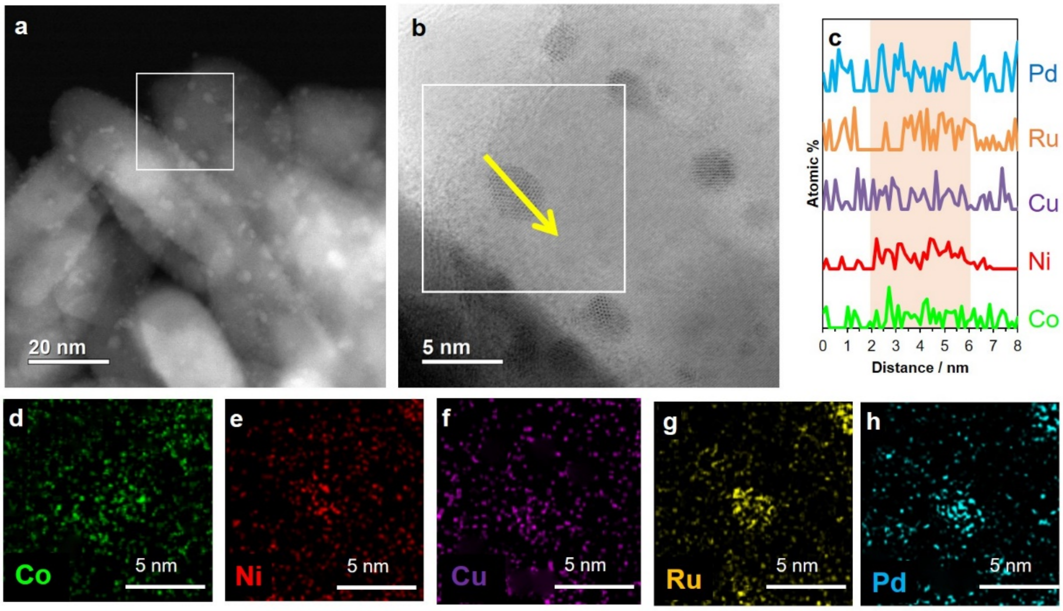

Supplementary Figure 16. (a) and (b) HAADF-STEM image of CoNiCuRuPd/TiO_2, (c) EDX line analysis along the arrow in (b), (d)-(h) EDX mapping of the corresponding elements.

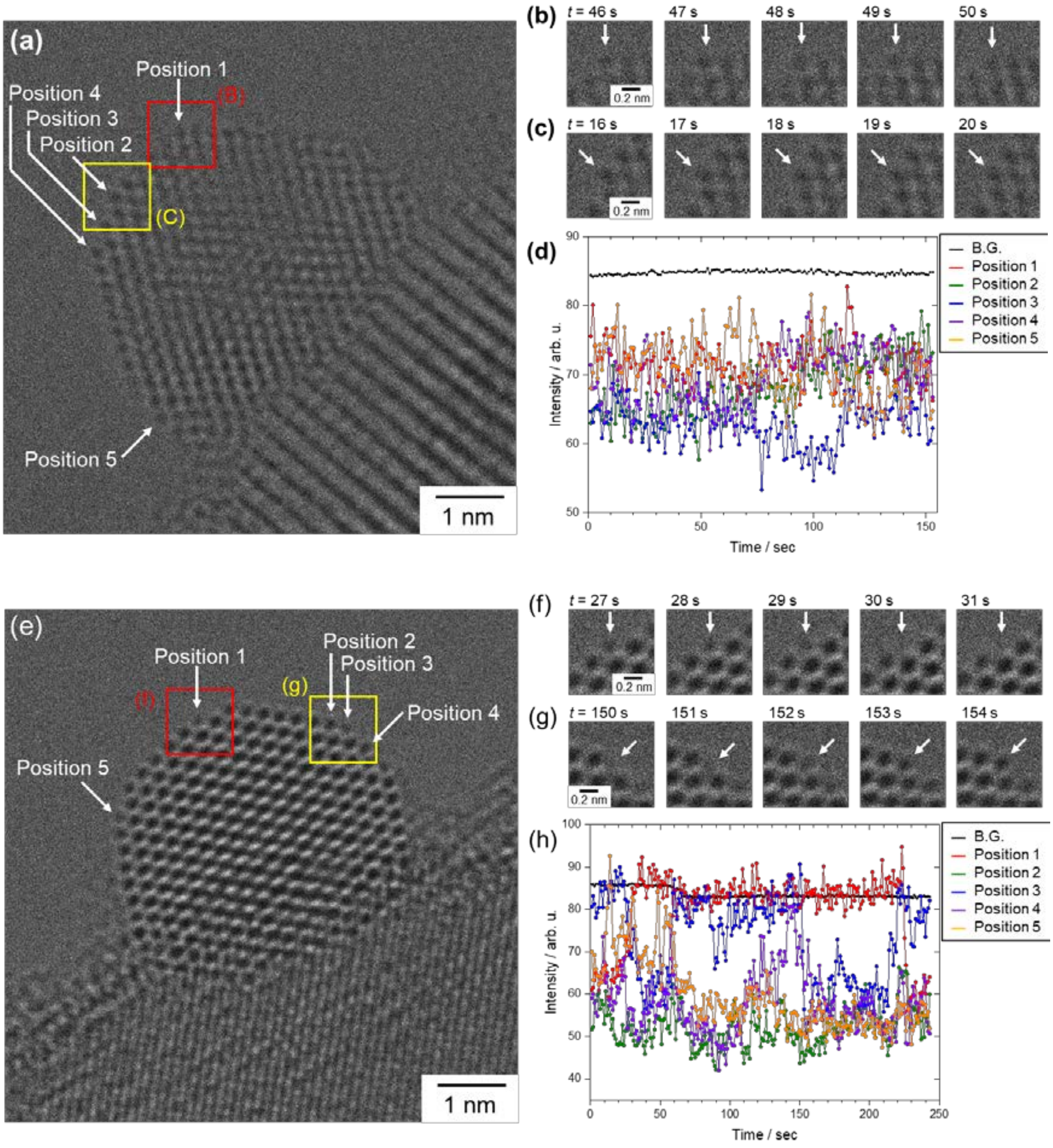

Supplementary Figure 17. Stability of surface atoms under electron beam irradiation of 2 A/cm^2 at 300 kV in vacuum using Titan ETEM G2 A representative TEM image of a nanoparticle in HEA/TiO_2 (a) and a Pd nanoparticle in Pd/TiO_2 (e). Sequential images of surface atoms in HEA/TiO_2 (b),(c) and Pd/TiO_2 (f),(g) taken from TEM movies (Supplementary Movies 1 and 2). Temporal changes in intensity at atomic columns in HEA/TiO_2 (d) and Pd/TiO_2 (h). The mean intensity at the regions (1.72 nm^2) without specimens was defined as background.

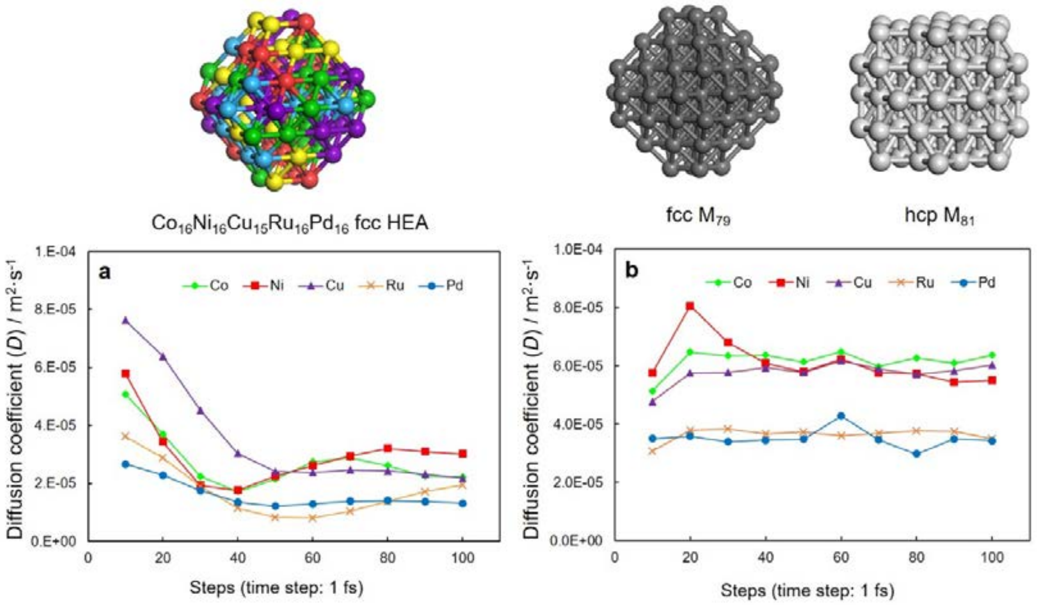

Supplementary Figure 18. Time profiles of the MD simulated atomic diffusion coefficients of (a) each element in the $Co_{16}Ni_{16}Cu_{15}Ru_{16}Pd_{16}$ fcc HEA, and (b) fcc M_{79} (M=Ni, Cu, Pd), and hcp M_{81} (M=Co, Ru) cluster models at 900 K (time step: 1 fs, and 100 steps).

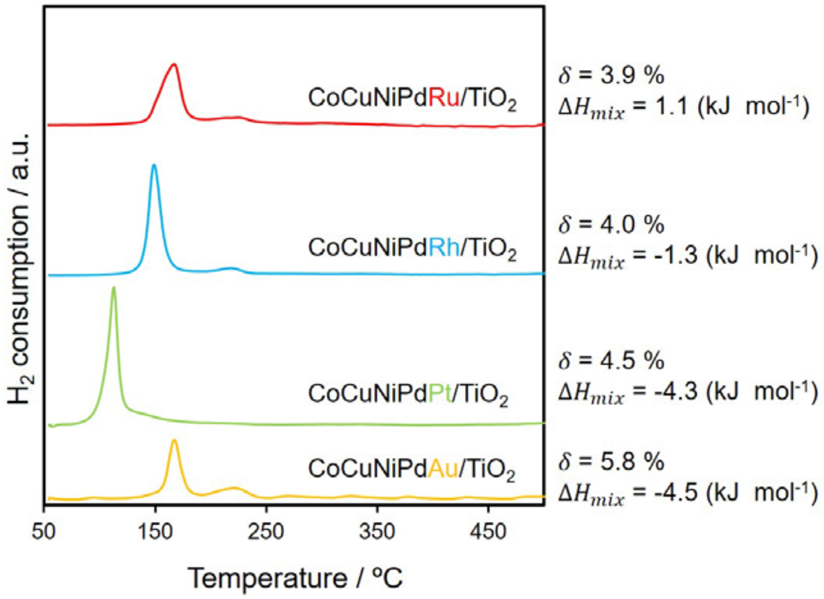

Supplementary Figure 19. H_2-TPR characterization of the reduction sequence for the as-deposited quinary-component samples supported on TiO_2. Such combination meet the required criteria the formation of a solid solution HEA (size difference, $\delta < 6.6\%$ and an enthalpy of mixing, -11.6 kJ/mol $< \Delta H_{\text{mix}} < 3.2$ kJ/mol).
