# Supplementary material for: Hydrogen spillover-driven synthesis of high-entropy alloy nanoparticles as a robust catalyst for CO2 hydrogenation
Source: Nat Commun. 2021 Jun 23;12:3884. doi: 10.1038/s41467-021-24228-z (PMC8222268; doi:10.1038/s41467-021-24228-z)
Supplement: Supplementary file 3 — Description of Additional Supplementary Files [file 41467_2021_24228_MOESM3_ESM.pdf]

Description of Additional Supplementary Files

File Name: Supplementary Movie 1
Description: TEM movie of a nanoparticle in HEA/TiO_2 under electron beam irradiation of 2 A/cm^2 at 300 kV in vacuum using Titan ETEM G2.

File Name: Supplementary Movie 2
Description: TEM movie of a nanoparticle in Pd/TiO_2 under electron beam irradiation of 2 A/cm^2 at 300 kV in vacuum using Titan ETEM G2.
